# Evaluating the Efficacy of Crushed Bictegravir/Emtricitabine/Tenofovir Alafenamide Administered via Tube

**DOI:** 10.1093/ofid/ofaf588

**Published:** 2025-09-24

**Authors:** Joshua Mercure, Kayla Bey, Eric Gillett, Jeffrey C Pearson, Suzanne M McCluskey, Alex E Rock

**Affiliations:** Department of Pharmacy, Massachusetts General Hospital, Boston, Massachusetts 02114, USA; Department of Pharmacy, Massachusetts General Hospital, Boston, Massachusetts 02114, USA; Department of Pharmacy, Brigham and Women's Hospital, Boston, Massachusetts 02115, USA; Department of Pharmacy, Brigham and Women's Hospital, Boston, Massachusetts 02115, USA; Division of Infectious Diseases, Massachusetts General Hospital, Boston, Massachusetts, USA; Department of Pharmacy, Massachusetts General Hospital, Boston, Massachusetts 02114, USA

**Keywords:** bictegravir, crushed, enteral administration, HIV, integrase inhibitors

## Abstract

Human immunodeficiency virus (HIV) viral suppression was evaluated after receipt of crushed or dissolved bictegravir/emtricitabine/tenofovir alafenamide (B/F/TAF) administered via enteral tube during hospitalization. Eighty-nine percent of patients (17/19) were virally suppressed (<200 copies/mL) within 1 year of receiving B/F/TAF via tube, suggesting that administration via tube is a reasonable alternative to changing antiretroviral regimens.

Bictegravir/emtricitabine/tenofovir alafenamide (B/F/TAF) is a widely prescribed, once-daily, single-tablet regimen (STR) approved for the treatment of HIV infection [1]. This regimen is often selected for its efficacy, high barrier to resistance, and favorable safety profile [2, 3]. However, B/F/TAF tablets are formulated to be swallowed whole, with no manufacturer recommendations regarding crushing or dissolving tablets. This presents a clinical challenge for hospitalized individuals who are unable to take medications by mouth, such as those with dysphagia or the need for mechanical ventilation.

Oral ingestion may be impractical in these scenarios, and considering the risk of developing resistance when disrupting antiretroviral therapy (ART) [4, 5], administering crushed or dissolved B/F/TAF through an enteral tube may be a preferable approach [6, 7]. Currently, there is limited clinical evidence regarding the efficacy of this strategy, and available data are restricted to in vitro studies or case reports.

The SOLUBIC trial was a phase I, open-label, randomized crossover study that analyzed the bioequivalence of B/F/TAF when administered whole, dissolved in water, or crushed in apple compote [8]. Results suggest that dissolving the medication in water demonstrates comparable bioequivalence to the administration of the whole tablet when examining AUC and C_max_ for each component of the STR. In contrast, administration of the crushed tablet did not achieve the same criteria for bioequivalence, raising the concern that this approach could precipitate acquired HIV drug resistance. Despite this, case reports describe favorable clinical outcomes when providing the tablet crushed through an enteral tube [9–11].

Further investigation is needed to evaluate the efficacy of alternative administration methods of B/F/TAF. Understanding the ability of this regimen to maintain viral suppression and prevent the development of resistance when given through alternative routes is essential for providing care in inpatient settings where oral administration is not feasible. This study aimed to assess virologic outcomes among hospitalized patients receiving crushed or dissolved B/F/TAF through enteral feeding tubes, providing clinical data for medication administration in this population.

## METHODS

This retrospective cohort study utilized electronic health record data from Massachusetts General Hospital and Brigham and Women's Hospital from February 2018 to December 2023. Individuals were considered for inclusion if their medication administration record data included B/F/TAF and had order routes listed as nasogastric, gastrostomy, orogastric, or jejunostomy tube during hospitalization. To better attribute sustained viral suppression to prolonged maintenance with a crushed or dissolved regimen, people with HIV (PWH) who received less than seven consecutive days of crushed or dissolved B/F/TAF or did not have HIV viral load (VL) data available within 12 months of crushed or dissolved B/F/TAF were excluded.

The primary endpoint was viral suppression, either new or sustained, during the designated 12-month follow-up period. Viral suppression was defined as an HIV RNA of < 200 copies/mL. Secondary endpoints included emergent HIV drug resistance and changes in ART regimens.

Patient characteristics and outcomes were analyzed using descriptive statistics, with continuous variables reported as medians with interquartile ranges (IQR) and categorical data reported as frequencies. All analyses were conducted using Microsoft Excel. The study protocol was approved by the Mass General Brigham Institutional Review Board.

## RESULTS

A total of 54 PWH were identified as having received crushed or dissolved B/F/TAF through an enteral tube during an inpatient hospitalization. Twenty-two PWH were excluded for receiving fewer than seven consecutive days of B/F/TAF, and 13 PWH were excluded for a lack of available HIV VL data within the 12-month follow-up period. A total of 19 patients were included in the final analysis.

The median age was 54 years [IQR 47.5–66.5], and 16 patients (84%) were male. Included individuals received B/F/TAF through an enteral tube for a median of 19 days [IQR 7.5–64]. Intubation was the primary reason documented as the need for administration through a tube in 14 patients (74%). Two individuals were documented as receiving dissolved B/F/TAF, and eight patients were documented as receiving the medication crushed. The method of administration for the remaining nine patients in the cohort was not described (Table 1).

**Table 1.** Patient Characteristics

Characteristic	Total Cohort (*N* = 19)
Age, years, median [IQR]	54 [47.5–66.5]
Male	16 (84)
Virally suppressed or undetectable prior to start	11 (58)
Resistance prior to crushed/dissolved B/F/TAF	8 (42)
INSTI	0 (0)
NRTI	5 (26)
NNRTI	4 (21)
PI	4 (21)
Development of resistance within 1 year	0 (0)
Duration of crushed/dissolved B/F/TAF, days, median [IQR]	19 [7.5–64]
Intensive care unit admission during hospitalization	16 (84)
Intubation as the reason for enteral tube	14 (74)
Crushed B/F/TAF for administration	8 (42)
Dissolved B/F/TAF for administration	2 (11)
Individuals with at least 1 missed dose	6 (32)
ART change during admission	2 (11)
Crushed/dissolved B/F/TAF continued after discharge	3 (16)

All data presented as *n* (%) unless otherwise stated.

Abbreviations: B/F/TAF, bictegravir/emtricitabine/tenofovir alafenamide; INSTI, integrase strand transfer inhibitor; IQR, interquartile range; NNRTI, non-nucleoside reverse transcriptase inhibitor; NRTI, nucleoside reverse transcriptase inhibitor; PI, protease inhibitor.

**Figure 1.**
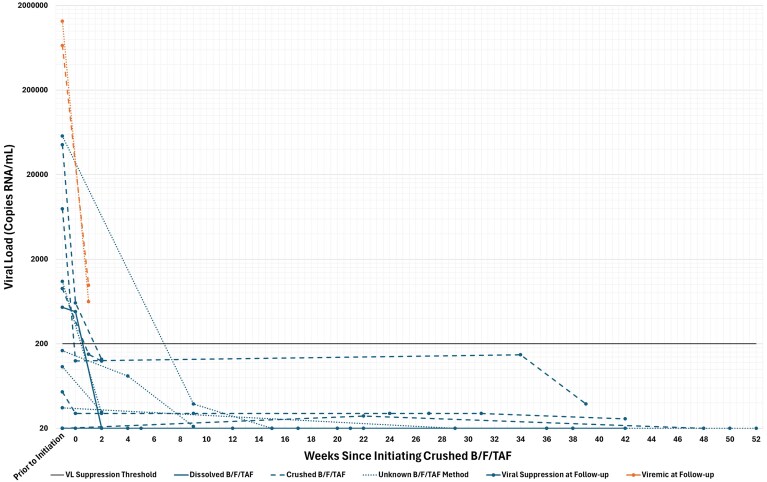
HIV Viral Load Testing within 1 year of Crushed B/F/TAF. HIV VL testing during a 12-month follow-up for 19 individuals that received a minimum 7 consecutive days of crushed or dissolved bictegravir/emtricitabine/tenofovir alafenamide (B/F/TAF) through an enteral tube. The VL suppression threshold is represented at 200 copies/mL with a solid black line. Orange lines indicate individuals who were viremic at their last recorded lab value, blue lines indicate individuals who were virally suppressed at their last recorded lab value. The modality of administration is indicated by line type, with dissolved, crushed, and unknown represented by solid, dashed, and dotted lines, respectively.

During the follow-up period, 17 patients (89%) achieved or maintained viral suppression. Among the eight patients with detectable viremia at hospital admission, six achieved undetectable VLs within the follow-up period. The two individuals who remained detectable after crushed or dissolved B/F/TAF administration still exhibited significant virologic response, as demonstrated by marked reductions in documented VLs, with a 2.8 and 3.3 log reduction, respectively (Figure 1).

Two patients underwent changes to their ART regimen during hospitalization. One patient was switched from B/F/TAF to limit drug-drug interactions with the initiation of empiric rifampin for tuberculosis treatment, and the other patient was switched to optimize their regimen in the setting of central nervous system escape and ongoing concerns for drug-drug interactions. Three patients continued administration of crushed or dissolved B/F/TAF after being discharged. Eight patients had documented resistance prior to the initiation of crushed B/F/TAF; no emergent resistance was identified in the cohort after 1 year based on available information in medical records.

There were eight deaths in the analyzed cohort, six of whom were admitted to critical care units during their admission. The leading cause of death was non-HIV-associated infection (*n* = 5). Two individuals died secondary to opportunistic infections associated with HIV, neither of which was viremic at the time of their passing. One death, a patient with hemorrhagic stroke, was unrelated to HIV, and the patient remained virally suppressed throughout their clinical course. No deaths were attributed to the administration of crushed or dissolved B/F/TAF.

## DISCUSSION

This study demonstrates that individuals who are unable to take medications by mouth and receive B/F/TAF when administered through an enteral tube in either a crushed or dissolved formulation can effectively achieve or maintain viral suppression. In our cohort, 89% of patients were virally suppressed during follow-up. All individuals who were viremic at the initiation of the crushed or dissolved formulation demonstrated significant reductions in their VL during the study, including the two individuals who remained viremic during the follow-up period. These results suggest that crushed or dissolved B/F/TAF presents an effective alternative when oral administration may not be practicable. This is particularly relevant for hospitalized individuals who may otherwise encounter an interruption in ART therapy or be forced to switch regimens.

Existing literature on the use of crushed or dissolved B/F/TAF, comprised largely of case reports, has also shown generally positive clinical outcomes with the maintenance of viral suppression using both crushed and dissolved administration of B/F/TAF [9–11]. Additional literature also describes this practice for other ART regimens, such as dolutegravir-based regimens [7, 12]. These findings, in addition to the favorable bioequivalence of dissolved administration described in the pharmacokinetic SOLUBIC study, suggest that this approach may be feasible in complex clinical scenarios [8].

One case report does note an increase in VL for an individual receiving crushed B/F/TAF through a percutaneous endoscopic gastrostomy tube [13]. They note no missed doses over the 37 days for which B/F/TAF was given and observed the emergence of the minor integrase mutation E157Q along with a VL increase from 5887 to 8047 copies during this time. Another similar report demonstrated virologic failure after 6 weeks of crushed B/F/TAF after an initially appropriate response over 4 weeks with the oral tablet [14]. They noted the development of resistance mutations M184V and R263K. Given these occurrences, close monitoring is recommended during longer periods of crushed or dissolved B/F/TAF, though no emergent drug resistance was identified in our larger study.

This study represents the largest available cohort in this patient population to date. The findings may have important implications for clinical practice. Administering B/F/TAF through a tube offers a practical and effective solution for maintaining viral suppression during periods when oral ART may not be feasible. Given that discontinuation or changes to suboptimal ART regimens can lead to virologic failure and resistance development, having a reliable method for continuing treatment with B/F/TAF could prevent these complications.

Our study has limitations. Specifically, the study used existing data from a single health system. The retrospective nature of the study design limits the ability to draw definitive conclusions without the ability to control for unmeasured confounders or undocumented information. Certain aspects may not be reflected in the data, such as adherence to regimens while outpatient and the full extent of comorbidities. Therapeutic drug monitoring was not performed in the study, so we cannot speak to the precise bioavailability and pharmacokinetics of crushed or dissolved B/F/TAF.

If tablets cannot be swallowed whole, B/F/TAF should ideally be given dissolved based on favorable bioequivalence data [8]. Recommendations from the SOLUBIC study are to flush the enteral tube with 30 mL of water before and after administering B/F/TAF, for which one tablet is dissolved in 240 mL of water, ensuring that the administration is separated from any enteral feeding by four hours to limit any interactions with polyvalent cations. However, the method of administration in our cohort was at the discretion of nursing staff during the study period, and crushed administration was common. Despite missing data that specified crushed versus dissolved administration in approximately half of the cohort, the prevalence of viral suppression at follow-up suggests that either approach may be clinically reasonable if enteral administration is otherwise impracticable. Additionally, individuals may have received B/F/TAF through a variety of methods, including nasogastric, gastrostomy, orogastric, or jejunostomy tubes. In some cases, multiple tube types were used for the same patient based on their clinical course. In these instances, the tube type with the most administration data was used for the analysis.

Future studies can aim to validate these findings in a larger, prospective manner to assess the long-term safety and efficacy of crushed or dissolved B/F/TAF. Therapeutic drug monitoring would allow for further insight into the pharmacokinetics of this approach.

In conclusion, the administration of B/F/TAF when given via tube led to the achievement or maintenance of viral suppression in the majority of the study population. The absence of emergent resistance and virologic failure following administration of B/F/TAF through an enteral tube in the inpatient setting is reassuring and suggests that providing B/F/TAF via tube is a reasonable alternative to changing or stopping ART altogether.
